# Dynamics of SARS-CoV-2 lineages in children and adults in 2021 and 2022

**DOI:** 10.1371/journal.pone.0316213

**Published:** 2024-12-20

**Authors:** Hiie Soeorg, Aare Abroi, Taavi Päll, Liidia Dotsenko, Erik Jaaniso, Katrin Kaarna, Andrio Lahesaare, Paul Naaber, Heiki Niglas, Ott Eric Oopkaup, Hedi Peterson, Tuuli Reisberg, Olga Sadikova, Steven Smit, Ulvi Gerst Talas, Radko Avi, Irja Lutsar, Kristi Huik

**Affiliations:** 1 Department of Microbiology, Faculty of Medicine, Institute of Biomedicine and Translational Medicine, University of Tartu, Tartu, Estonia; 2 Infection, Immunity and Inflammation Research & Teaching Department, Great Ormond Street Institute of Child Health, University College London, London, United Kingdom; 3 Faculty of Science and Technology, Institute of Technology, University of Tartu, Tartu, Estonia; 4 Department of Communicable Diseases, Health Board, Tallinn, Estonia; 5 Faculty of Science and Technology, Institute of Computer Science, University of Tartu, Tartu, Estonia; 6 Clinical Research Centre, Faculty of Medicine, Institute of Clinical Medicine, University of Tartu, Tartu, Estonia; 7 Tartu University Hospital, Tartu, Estonia; 8 SYNLAB Eesti OÜ, Tallinn, Estonia; 9 High Performance Computing Center, Faculty of Science and Technology, Institute of Computer Science, University of Tartu, Tartu, Estonia; 10 Faculty of Science and Technology, Institute of Genomics, University of Tartu, Tartu, Estonia; Carol Davila University of Medicine and Pharmacy, ROMANIA

## Abstract

**Purpose:**

We aimed to describe SARS-CoV-2 lineages and diversity in children and adults in Estonia and similarity to travel-related cases and neighbouring countries.

**Methods:**

SARS-CoV-2 sequences in 2021–2022 from a nationwide study were included. The proportion of predominant lineages in Estonian regions and among travel-related cases was described by multinomial logistic regression. Simpson’s indices of diversity were compared using linear regression. Dynamics of Bray-Curtis dissimilarity was described by applying fuzzy clustering to non-metrical dimensional scaling results.

**Results:**

A total of 2,630 sequences from children (<15 years) and 23,031 from adults (≥15 years) were included. The increase in the proportion of Alpha/Delta/Omicron BA.1/BA.2 lineages was delayed in smaller regions (by 3.5–27.5 days). The proportion of Alpha/Delta/Omicron BA.1 increased earlier among travel-related (n = 4,654) than non-travel-related cases (10.5 days). Diversity was lower in non-travel-related than travel-related cases until Delta period by 0.066. Dynamics of lineages and diversity were similar in adults and children. Similarity of lineages was delayed compared to Finland during Alpha/Omicron BA.1/BA.2 periods and different from all neighbouring countries during Delta period.

**Conclusion:**

SARS-CoV-2 lineages in children and adults were similar. Differences between regions and travel-related cases and varying similarity to neighbouring countries suggest the importance of mobility in the spread.

## Introduction

SARS-CoV-2 infection in children differs from that in adults in several aspects–children have longer incubation time [1], lower attack rates for the infection with Alpha variant [2], and are more likely asymptomatic infection with Omicron variant [3]. These factors may have an impact on the spread of infection. In addition, age group [4, 5] and social activity [6] are important factors that determine the spread of an infectious agent in a population. This is exemplified by the increased spread of the virus after re-opening schools during the pandemic [7]. As host migration is one of the most important determinants of infection establishment [8] and mobility patterns vary between regions, i.e., some regions are better connected [9], as well as social activities are region-specific [6], the geographical region should be considered in describing the spread.

Few studies have described SARS-CoV-2 lineages from adults and children and shown the close relationship of samples from children and adults in the phylogenetic tree [10] or initial dissemination of Omicron’s sub-lineage BA.2 in the younger group [11]. However, no studies have compared lineages in children and adults over a longer period. Understanding the distribution of lineages that reflects the spread of SARS-CoV-2, may serve as an important source of information on the impact of policies to reduce the spread of viruses [12]. As longitudinal studies comparing lineages in adults and children are lacking, the possible impact of the characteristics of the virus, restrictions and immunity, which changed during the pandemic, to the spread of the virus has not been fully understood [13].

We aimed to describe the dynamics of SARS-CoV-2 lineages and genetic diversity in children and adults in Estonia, stratified by different Estonian regions. Second, we aimed to describe the similarity of Estonian cases with those from neighbouring countries and the effect of cross-country mobility on the spread of SARS-CoV-2 lineages by comparing the Estonian cases with travel-related cases.

## Materials and methods

### Study data

This analysis is part of a nationwide SARS-CoV-2 whole genome sequencing study conducted (sample collection preformed) from 13 March 2020 to 31 March 2023 in Estonia [14]. For that study, the Estonian Health Board carried out randomly selected sampling of SARS-CoV-2 PCR-positive tests according to the epidemiological needs in managing the epidemic and local outbreaks. The random sampling was performed proportionally to population size, age and gender distribution in each region, i.e., it a stratified random sampling. From local outbreaks, in general two to five samples were selected for sequencing to keep the effect of outbreaks on minimum level. We included the samples randomly selected for sequencing from SARS-CoV-2 PCR-positive persons proportionally to regional population size, age, and gender between February 1, 2021, and October 30, 2022 (when free PCR testing was cancelled). Additionally, all travel-related cases (SARS-CoV-2 PCR-positive samples from people who had visited a foreign country within the last 14 days) from February 7, 2021, were included except from January 6 to January 15, 2022, when due to the high number of travel-related cases, 41% of all cases were sequenced. Between February 1 and 6, 2021, out of 56 travel-related cases 15 were included.

For sequencing of paired-end 75 bp or 150 bp reads using the Illumina NextSeq500, viral RNA was reverse transcribed and amplified by the Illumina COVIDSeq Test Kit Artic v3 or v4/v4.1 (Illumina Inc, San Diego, CA, USA). A proportion of samples were sequenced by Eurofins Genomics Germany GmbH using in-house method similar to the ARTIC primers (this was brokered by the ECDC). Detailed sequencing is described in Päll *et al*. 2024 [14]. The lineage designation was carried out using Pangolin UShER v1.14, in some cases scorpio-only mode was used with SCORPIO v0.1.10.

Only samples with known age and county of the SARS-CoV-2 PCR-positive person were included in the final analysis. Those with age <15 years are hereafter referred to as children, and those ≥15 years as adults. For the analyses, Estonia (S1 Fig in S1 File) was divided into four regions (three largest in terms of population size and the remaining): Harju, where the capital Tallinn is located and which is the largest town and an important traveling hub for Estonians with airport and seaport; Tartu, where the largest university in Estonia is located and that is well connected with the capital; Ida-Viru, the third largest county is Estonia, located at the border of Russia, where majority is Russian-speaking people; Other, the remaining counties.

Lineage information of SARS-CoV-2 spreading in the neighbouring regions of Estonia during the study period were obtained from GISAID [15]. For that, the metadata of sequences found using search restrictions ‘Complete’ and ‘Low coverage excluded’ on October 25 (for St. Petersburg and Leningrad Oblast using different spellings of the city names), October 31 (Latvia) or November 10, 2023 (Finland) was downloaded. Pango lineages of SARS-CoV-2 from samples with known collection date were used for analysis, as described below.

The study was approved by the Research Ethics Committee of the University of Tartu (approvals 304/T-1, 324/T-1, 376/M-7). Informed consent was not required based on § 6 of the national ‘Personal Data Protection Act’.

### Statistical analysis

#### Difference in the proportion of sequences

For determining time periods when the proportion of sequenced cases was statistically significantly different between children and adults, the method by Bai and Perron *et al*. [16] implemented in R package strucchange [17] was used to determine the dates that separate structural changes in the linear model of the difference between the proportions.

#### Differences between regions

The weekly proportion of lineages over time in the four regions was described with multinomial regression analysis [18]. Those lineages that comprised >5% of all sequences in at least one week in all four regions were included separately and the remaining were categorised as Other lineages. The inclusion of interaction between region and time (included as natural cubic splines with the knot at the median), and age group (children vs adults) and time was tested. Akaike Information Criterion (AIC) was used to select the final model. Due to the complexity of the interpretation of multinomial logistic regression model coefficients, predicted probabilities from the model were used for the presentation of the modelling results [19]. Subsequently, the following parameters were derived to test the difference in the spread of variants between regions: maximum growth rate (the value of the steepest slope of the time-proportion curve of a lineage), maximum proportion (the value of the highest weekly proportion of a lineage achieved) and timing of maximum growth rate (the week when the maximum growth was achieved) relative to the earliest location, as described earlier [18]. Differences in these parameters between the regions were tested using linear regression (after log10-transformation), beta regression and negative binomial mixed effects model, respectively, where the lineage was included as random effect and region, variant and their interaction were tested as explanatory variables. Delta method was used to calculate the standard errors and subsequently 95% confidence intervals of transformations of model coefficients.

#### Travel-related cases

The difference in the distribution of lineages each week between travel-related cases from adults and children was tested using Fisher’s exact test and p-values were adjusted using Bonferroni correction. The weekly proportion of lineages over time among travel-related cases compared with the cases in the two largest regions in Estonia was described with multinomial regression analysis. Subsequently, the same parameters were obtained and compared, as in the case of comparison between regions of Estonia. Those lineages that comprised >5% of all sequences in at least one week in both sets were included separately and the remaining were categorised as Other lineages.

#### Genetic diversity

For genetic diversity, weekly Simpson’s diversity index of lineages was calculated. Simpson’s diversity index shows the probability that two randomly selected SARS-CoV-2 viruses from a given set do not belong to the same lineage [20]. For determining time periods when genetic diversity was significantly different between the four regions, children and adults, and cases in Estonia and travel-related cases, breakpoints that separate structural changes in linear models of the differences between Simpson’s diversity indices in two compared datasets were estimated using the method by Bai and Perron *et al*. [16] implemented in R package strucchange [17]. To reduce the uncertainty in the estimation of Simpson’s index of diversity, only weeks when the number of samples was at least ten were retained; for the other weeks, the estimate was imputed using Structural Model and Kalman smoothing [21]. The linear models of the differences between Simpson’s diversity indices included lagged difference as an explanatory variable to correct for serial correlation.

#### Dissimilarity

Bray-Curtis dissimilarity indices (the sum of differences in the proportions of lineages, each difference standardized by the sum of the proportions of the lineage [20]) between weekly lineage distributions in Estonian adults, children and travel-related cases, and Finland, Latvia and St. Petersburg were calculated for all weeks with at least ten sequences. Non-metric multidimensional scaling (NMDS) was applied to the dissimilarity matrix to reveal possible convergence or divergence of the weekly lineage distributions. As rapid lineage turnover leads to saturation of the dissimilarity index and results in the horseshoe effect and consequently misinterpretation, the Local Manifold distance algorithm [22] was used to adjust dissimilarity measures to represent true ecological distances between samples better. To further reduce the effect of rapid lineage turnover on NMDS, the observation period was divided into periods when variants predominated (comprised at least 50% of all lineages). Fuzzy clustering was applied to NMDS dimensions to detect possible lags in similarity between countries by observing transitions from one cluster to another. Fuzzy silhouette index was used to determine the number of clusters. A cluster includes weeks during which the distribution of lineages that spread were more similar to each other than to the weeks in other clusters. One cluster could contain weeks from several countries.

#### Time series model

Autoregressive integrated moving average (ARIMA) model with regressors was used to determine factors related to Bray-Curtis dissimilarity index and Simpson’s genetic diversity index values between adults and children. For the weeks when there were fewer than ten samples, the diversity and dissimilarity estimate was imputed using Structural Model and Kalman smoothing [21]. The variables tested as regressors were the stringency index, government response index, containment and health index, school closures from a global panel database of pandemic policies (Oxford COVID-19 Government Response Tracker) [23] and school holidays and their up to 4-week lags, and Simpson’s diversity index (in case of Bray-Curtis dissimilarity). From the Oxford COVID-19 Government Response Tracker, daily values of indices and whether schools were open or closed in Estonia were obtained and weekly averages calculated for the time series analysis. Arcsine square root transformation was applied to the indices prior to the fitting of models. The stationarity of the time series was tested using the Kwiatkowski, Phillips, Schmidt and Shin test. AIC was used to determine ARIMA orders. ARIMA model was accepted if the Ljung-Box test indicated no autocorrelation. AIC and statistical significance of the coefficients of regressors were used to determine the final model.

## Results

During the study period, a total of 558,034 PCR-positive Estonian SARS-CoV-2 cases were detected, of which 16.1% (n = 89,834) were from children and 83.9% (n = 468,200) from adults. A total of 25,661 samples (4.6% of all Estonian cases) were sequenced and included in the analysis. Of these, 10.2% (n = 2,630) were from children and 89.8% from adults (n = 23,031). Of cases in children, 2.9% were sequenced, and of cases in adults, 4.9%, with the proportion varying over time but being statistically significantly different only at the beginning of Delta period and during Omicron BA.5 period (S2 Fig in S1 File), when the incidence was small in both groups (S4 Fig in S1 File). A total of 321 different lineages were detected, 113 in children (9 only in children–AY.4.2.3, AY.43.2, AY.70, BA.1.1.15, BA.1.21, BA.1.9, BA.2.71, BA.5.8, BN.1) and 312 in adults (208 only in adults).

Of all sequences, 46.4% were from Harju (n = 11,917), 11.6% from Tartu (n = 2,964), 10.4% from Ida-Viru (n = 2,657) and 31.6% from other counties (n = 8,123). A total of 14 lineages were detected in >5% of samples in at least one week in all four regions: AY.100 (n = 964, 3.8% of all sequences), AY.122 (n = 3436, 13.4%), AY.122.2 (n = 598, 2.3%), AY.128 (n = 318, 1.2%), AY.4.5 (n = 411, 1.6%), AY.43 (n = 570, 2.2%), B.1.1.7 (n = 4884, 19.0%), BA.1 (n = 559, 2.2%), BA.1.1 (n = 1213, 4.7%), BA.2 (n = 3493, 13.6%), BA.2.9 (n = 1222, 4.8%), BA.5.1 (n = 762, 3.0%), BA.5.2 (n = 1276, 5.0%), BA.5.2.1 (n = 494, 1.9%).

**Fig 1:**
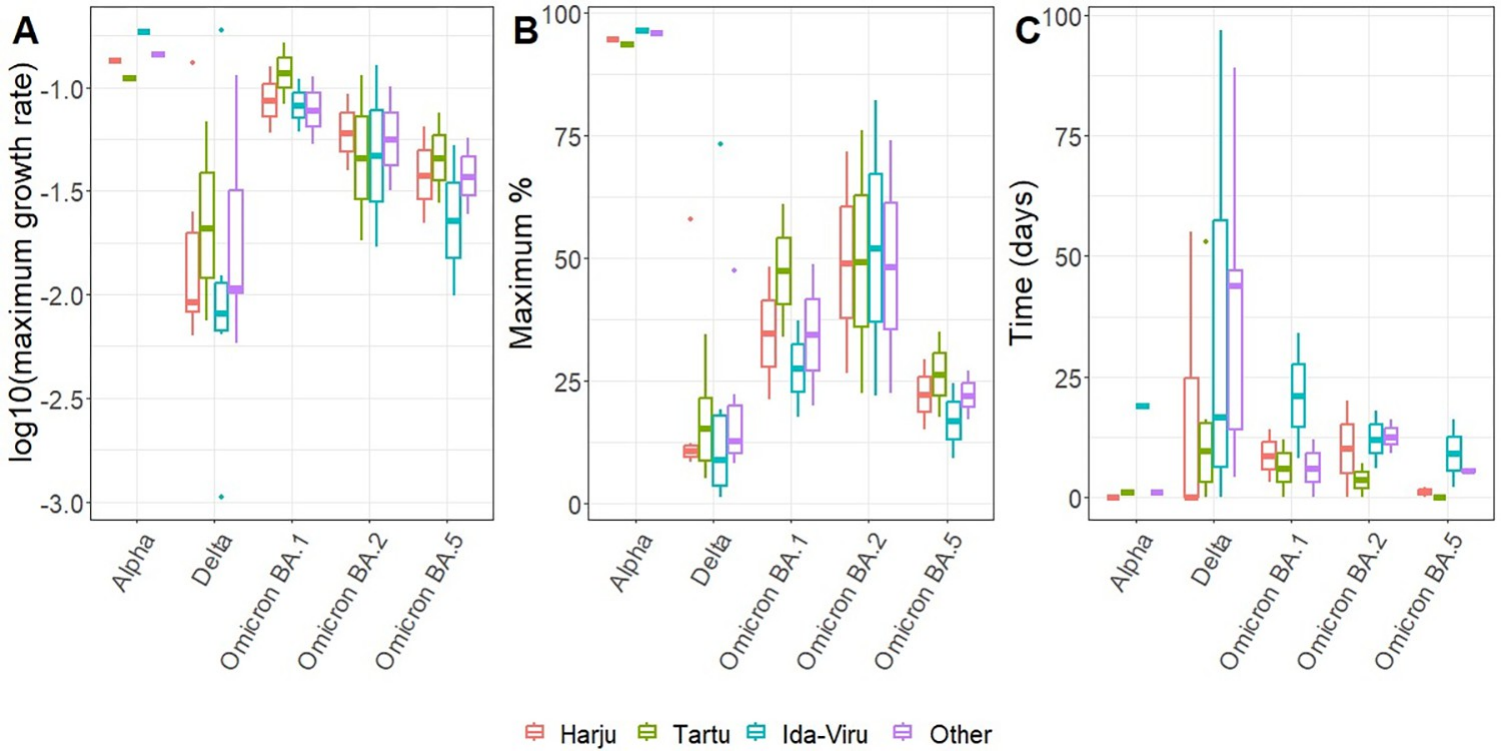
Parameters describing the spread of SARS-CoV-2 in different regions. Boxplots of parameters derived from the final multinomial regression model by variants and regions: (A) log10-transformed maximum growth rate of lineages, (B) maximum proportion, (C) time of maximum growth rate relative to the location where the maximum growth rate was reached the earliest. From the models of derived parameters, BA.5.2 was omitted as the time when it reached its maximum proportion was estimated to be in the last week of the observation period, possibly leading to incorrect estimates.

The final multinomial regression model of the weekly proportions of different lineages included interaction between natural cubic splines of time and the four-level region, showing that dynamics of the proportion of at least one lineage differed between the regions. The age group did not improve the model. The fit of the final model to the data is shown in S3 Fig in S1 File. Log10 of maximum growth rate was the lowest in the case of lineages of Delta variant (on average by -0.6 (95% CI -1.0…-0.2)) compared with other variants and in Ida-Viru (-0.2 (-0.3…-0.03)) compared with other counties (Fig 1A). Lineages of Alpha variant reached higher proportion (93.9% (95% CI 75.1…98.8%)) compared with Delta (15.5% (7.1…23.8%)), Omicron BA.1 (35.0% (10.7…59.4%)), Omicron BA.2 (49.8% (22.9%…76.6%) and Omicron BA.5 (21.7% (3.3…40.1%) (Fig 1B). No difference in proportion between counties was detected. Maximum growth was delayed in Ida-Viru and Other regions compared with Harju and Tartu by 27.5 days (0.2…54.8) in the case of lineages of Delta variants, 11.5 days (-1.3…24.2) in the case of Omicron BA.1 and BA.2 variant, and 3.5 days (95% CI 0.04…6.9) in the case of Alpha and Omicron BA.5 variants (Fig 1C).

A total of 4,654 cases were travel-related, 334 in children and 4,320 in adults. The number of travel-related cases peaked prior to the incidence peaks corresponding to Delta and Omicron BA.1-BA.2 spread but was delayed compared to the incidence peak corresponding to Alpha spread (S4 Fig in S1 File). A total of 140 lineages were detected among travel-related cases, 55 in both adults and children, 83 only in adults, and 2 only in children (B.1.637, BA.1.1.2). There was no statistically significant difference in the overall distribution of lineages between children and adults. Of all these lineages, 46 were not detected among non-travel-related cases, which comprised altogether 1.8% of travel-related cases (82 of 4,654). Including only random samples during the period when at least one travel-related case was sequenced, of a total of 133 lineages, 40 were not detected among travel-related cases which represented 0.5% of the sequenced samples (96 of 19,339).

**Fig 2:**
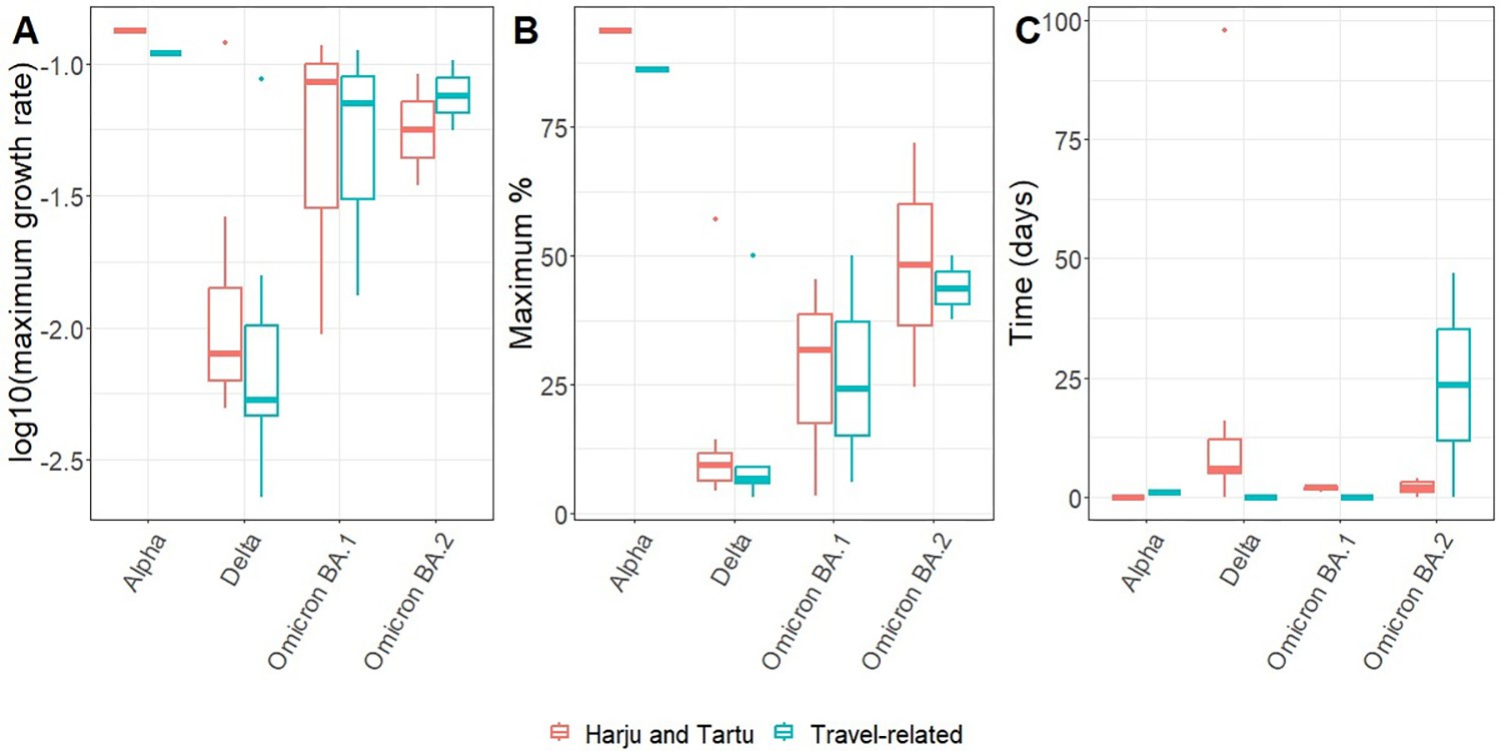
Parameters describing the spread of SARS-CoV-2 among travel-related and non-travel-related. Boxplots of parameters derived from the final multinomial regression model of the proportion of most common lineages among travel-related cases and non-travel-related cases in the two largest regions in Estonia (Harju and Tartu) by variants and origin: (A) log10-transformed maximum growth rate of lineages, (B) maximum proportion, (C) time of maximum growth rate relative to the location where the maximum growth rate was reached the earliest. From the models of derived parameters, B.1.1, B.1.1.317, B.1.1.7, B.1.177.60, B.1.221 and B.1.258 were omitted as the time when it reached its maximum proportion or maximum growth rate was estimated to be in the first week of the observation period, possibly leading to incorrect estimates.

Twenty different lineages were included in the multinomial regression model of the weekly proportions of different lineages among travel-related cases and non-travel-related cases in the two largest regions in Estonia (Harju, Tartu) over time. The final model included interaction between natural cubic splines of time and whether the case was travel-related, showing that dynamics of the proportion of at least one lineage differed between the non-travel and travel-related cases. The age group did not improve the model. The fit of the final model to the data is shown in S5 Fig in S1 File. Log10 of maximum growth rate was lower in the case of lineages of Delta (by -0.8 (95% CI -1.2…-0.4)) compared with other variants (Fig 2A), but there was no difference between travel-related cases and non-travel-related cases. Lineages of Alpha variant reached a higher proportion (90.5% (95%CI 74.9…100.0%)) compared with Delta (9.9% (4.4…15.3%)), Omicron BA.1 (21.9% (4.3…39.5%)) and Omicron BA.2 (45.8% (15.1%…76.4%) (Fig 2B). No difference between the proportion of lineages from travel-related and non-travel-related cases was detected. Maximum growth was delayed in the two largest regions in Estonia compared with travel-related cases by 10.5 days (-3.6…24.6) in the case of lineages of Alpha, Delta and Omicron BA.1 variants but earlier in the case of Omicron BA.2 variant by 22.8 days (-22.2…67.7) (Fig 2C).

**Fig 3:**
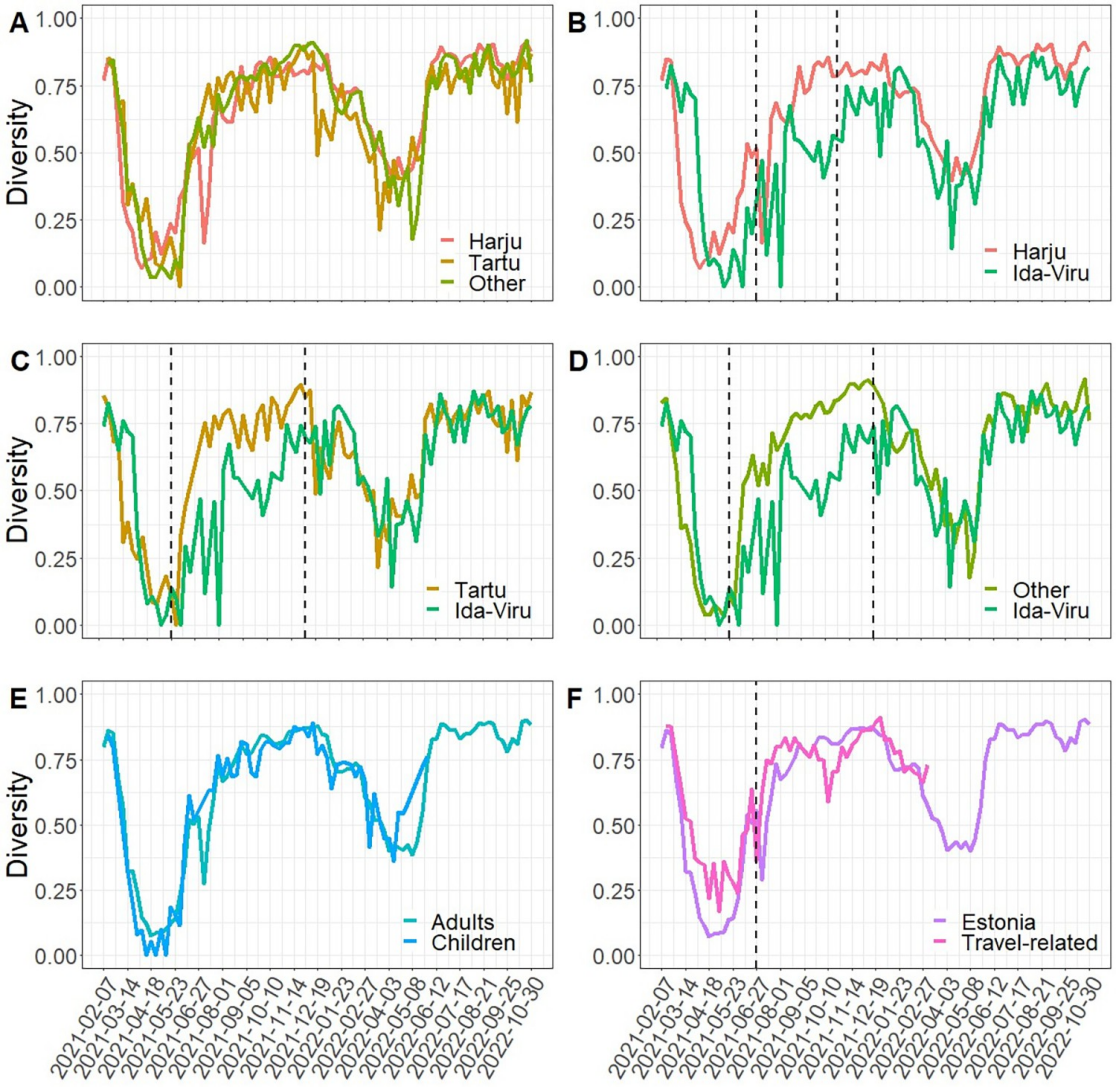
Simpson’s genetic diversity indices. Simpson’s genetic diversity index over time in (A) Harju, Tartu and Other counties between which no differences were detected, (B)-(D) Harju, Tartu and Other, respectively, vs Ida-Viru county where differences between weeks marked by vertical dashed lines were detected, (E) children and adults, (F) travel-related and non-travel-related cases in Harju, Tartu and Other counties where difference before week marked by vertical dashed lines was detected.

Genetic diversity was significantly lower in Ida-Viru compared with the other three regions during the spread of Delta virus (May or June 2021 until October or December 2021) and greater in Ida-Viru compared with Other regions until the spread of Delta virus (February to May 2021) (S1 Table in S1 File) (Fig 3A–3D).

There was no difference between the genetic diversity of children vs adults when data from Ida-Viru were excluded (difference of -0.002 (standard error 0.009) (p-value 0.8) during 01.02.2021–29.05.2022; later <10 samples were available from children each week) (Fig 3E). Travel-related cases had higher genetic diversity compared to non-travel related cases excluding Ida-Viru by 0.07 (0.03) (p-value 0.02) between 01.02.2021–27.06.2021, but not different thereafter until 06.03.2022 (after that <10 travel-related cases were sequenced) (difference of -0.02 (0.02), p-value 0.4) (Fig 3F).

**Fig 4:**
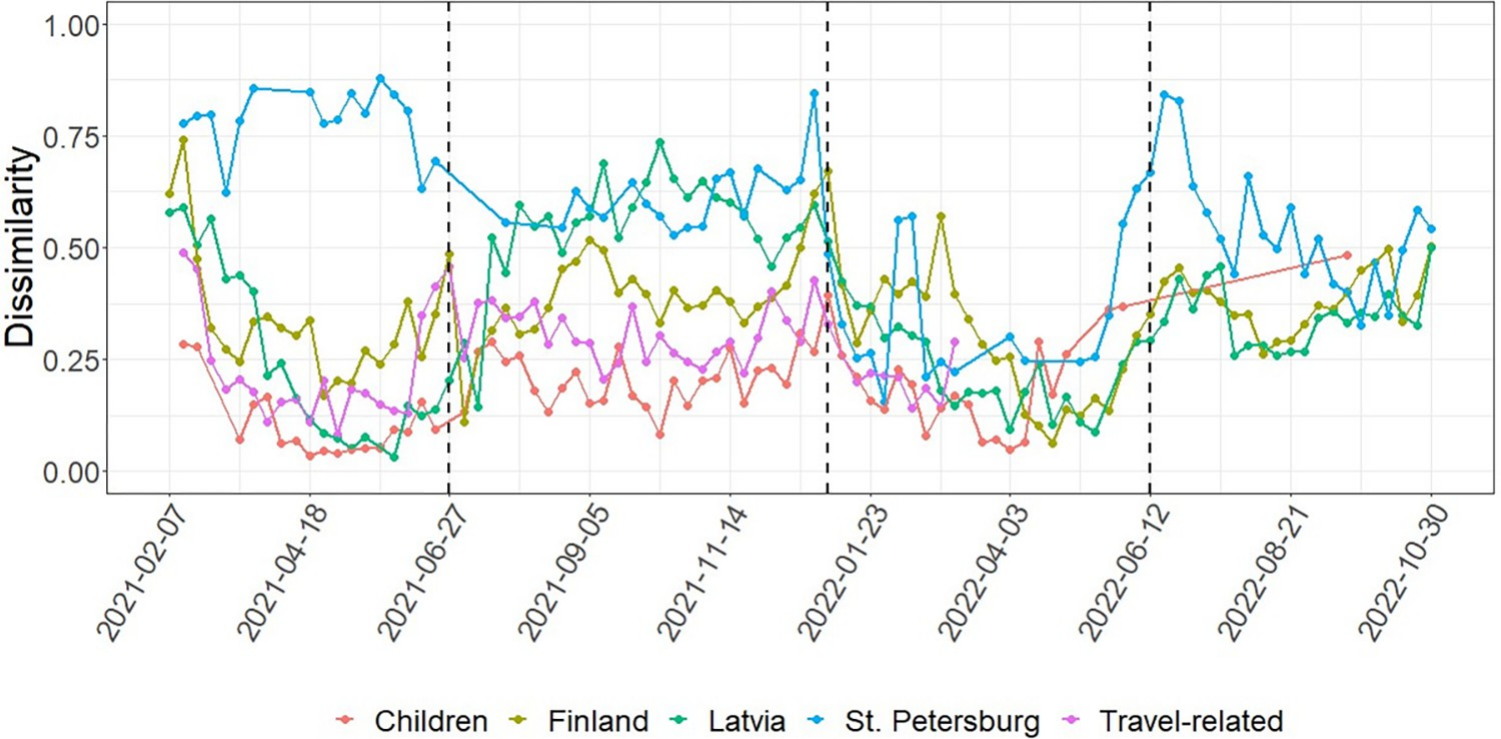
Bray-Curtis dissimilarity indices. Bray-Curtis dissimilarity index over time between weekly lineage distributions in Estonian adults and Estonian children, travel-related cases, Finland, Latvia and St. Petersburg. Dashed vertical lines delineate the periods of the predominance of Alpha, Delta, Omicron BA.1 or BA.2 and Omicron BA.5 variant.

A total of 39,184 sequences from Finland, 24,818 from Latvia and 5,629 from St. Petersburg were obtained. Overall, according to Bray-Curtis dissimilarity index, lineages in Estonian adults were most similar to the Estonian children, followed by travel-related cases (Fig 4). During Alpha predominance period, lineage distribution in Estonia was more similar to that in Latvia, thereafter during Delta predominance period to that in Finland, and during the first week of Omicron spread more similar to Latvia. Lineages in St. Petersburg were the least similar to Estonian adults and children.

**Fig 5:**
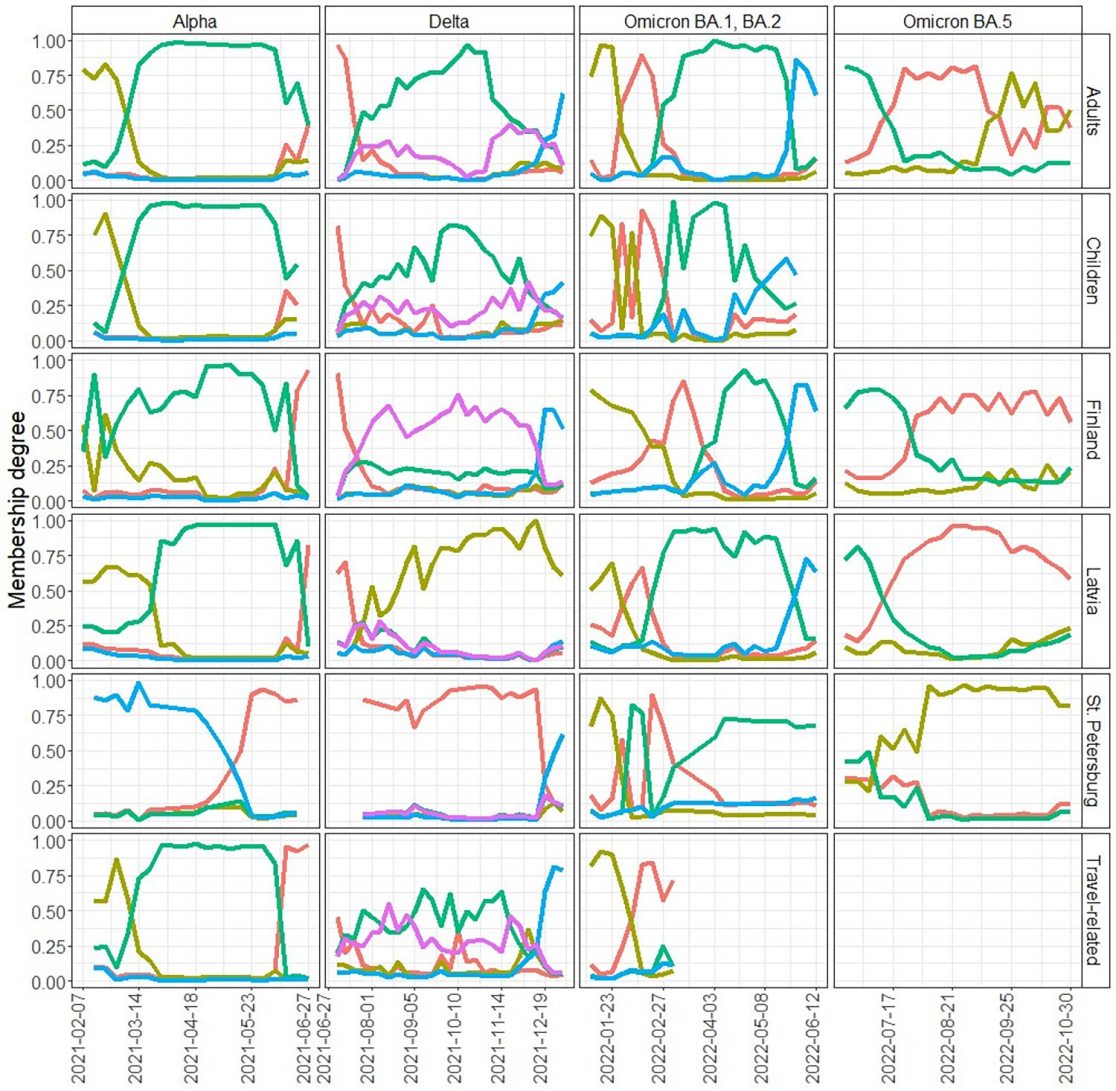
Fuzzy clustering of non-metric multidimensional scaling results. Membership degrees of weekly lineage distributions of Estonian adults, children and travel-related cases, and Finland, Latvia and St. Petersburg according to fuzzy clustering applied to non-metric multidimensional scaling results of Bray-Curtis dissimilarity matrix. Colours indicate the same cluster only in case of the same time period, i.e., in the same column.

Fuzzy clustering of NMDS dimensions revealed three clusters during Omicron BA.5 period, four clusters during Alpha and Omicron BA.1, BA.2 periods and five during Delta period. Fig 5 shows how the degrees of membership changed over time. The first two dimensions of NMDS are shown in S6-S9 Figs in S1 File. During most of the Alpha period, all Estonia weeks belonged to the same cluster with Finland and Latvia. During the spread of Delta, Estonia clustered separately from Latvia and Finland. During the spread of Omicron BA.1 and BA.2, cluster memberships of Estonia, Latvia and Finland varied over time in a similar pattern. During Omicron BA.5 period, the clustering of Estonia, Latvia and Finland was similar. St. Petersburg clustered during the whole observation period into different clusters, apart from during the spread of Omicron BA.1 and BA.2. Fuzzy clustering results are in line with weekly lineage distributions shown in S10-S13 Figs in S1 File for all countries.

According to ARIMA with regressors, only Simpson’s genetic diversity index in the same week was associated with dissimilarity, increasing dissimilarity by 0.1 (95% CI 0.1–0.1). No tested factor was associated with genetic diversity.

## Discussion

In this study, we showed that the spread of predominant SARS-CoV-2 lineages, genetic diversity and similarity to neighbouring countries in terms of the spreading lineages was similar in children and adults in Estonia. Regional differences within Estonia and between Estonia and neighbouring countries as well as travel-related cases show once again that dispersal patterns of the virus varied during the pandemic [24].

In other respiratory infections, children have a higher incidence [25] and thus have a major role in the spread. In this study on SARS-CoV-2, we did not find differences in the spread between adults and children. According to findings from other studies, differences in the spread of lineages might have been plausible. First, children have a decreased risk of infection compared with adults [2, 26, 27], which may facilitate earlier establishment of infection among adults. Second, the higher incidence of SARS-CoV-2 infection since Delta period [26] in children compared with adults and the increased risk of infection in households with children [28] underlines the role of younger age groups in spreading the virus. However, these effects may have been offset by higher immunity by earlier vaccination in adults, which decreases the susceptibility to the infection [29], and a larger number of sequences in adults, which might have facilitated earlier detection of lineages with low number of cases during the initial period of their spread. Nevertheless, the lack of differences between children and adults could have arisen in considerable part just from the extensive mixing of the two populations. Transmission of SARS-CoV-2 occurs the best in households, compared with, e.g., school setting [30], whereas children are as likely as adults to transmit the virus [31]. Although schools may serve as places where children facilitate the spread of the virus among them due to a large number of contacts between students, teachers have a higher risk of transmission [32, 33] and this may level out the possible impact of such difference on the extent of spread among children compared with adults. Still, the dissimilarity index showed a slight difference in the lineage distribution between children and adults. This can be attributable to random variation in the sampling of the many lineages spreading, shown by the variation in similarity related to genetic diversity, which is expected, as Bray-Curtis index partly decomposes into Simpson’s index [34]. Furthermore, the larger the similarity, the more it is underestimated, especially in the case of small samples [35]. Although local outbreaks in children occur, and probably also did in Estonia, occasionally earlier than the spread among adults is detected [11], our results show no major differences between adults and children.

Similarly to other countries [36], we found some differences in the spread of lineages between counties. Mobility is an important determinant of how fast a strain spreads to a location [9]. In line with that, as Harju and Tartu are the most well-connected places in Estonia, the even spread of the strains to these regions was expected. Despite fewer connections between Other regions and Harju and Tartu, no differences in genetic diversity were seen between these regions. Rural areas are important sources of evolution and transmission of SARS-CoV-2 [37], which might be particularly true in our case, as in regions outside the Harju, Tartu and Ida-Viru, by the beginning of our study, the seroprevalence was the lowest [38], facilitating the spread of new strains. Furthermore, there was an increase in migration to non-metropolitan rural areas during the pandemic among Estonians [39]. Lower genetic diversity in Ida-Viru may be partly attributable to the very high infection rates in spring 2021 compared to the other parts of Estonia [40], as higher immunity levels reduce diversity [41]. This recent higher disease burden may also explain the lower transition speed in Ida-Viru, possibly due to elicited protective immunity [18]. Additionally, the lower connectedness of Ida-Viru with other regions in Estonia may be related to regional differences in lineage diversity [36] and within-region encapsulation and modest contacts between communities [42] could have contributed to slower spread of the virus. Although Simpson’s genetic diversity index is relatively robust to undersampling [43], we cannot exclude its effect as Ida-Viru had the smallest number of samples sequenced.

Travel-related cases occurred earlier than the non-travel-related cases in Estonia before the arrival of Omicron BA.2. This is expected, as travel is an important source of SARS-CoV-2 variants [24]. A similar importation lag has been described earlier [36]. Later detection of cases of Omicron BA.2 variant compared with non-travel-related cases can be partly explained by its rapid spread. More extensive spread, shown by the very high incidence of the infection during the spread of Omicron BA.2, can be more rapidly detected in the country [36] and the assumption that a travel-related case was acquired abroad may not hold [44]. Still, we cannot exclude inaccurate estimates due to the small number of travel-related cases reported during Omicron BA.2 spread. The similarity of lineages in Estonia to those in neighbouring countries varied in line with the travel restrictions, further underlining the importance of mobility. At the beginning of Alpha period, Finland banned travellers from high-risk regions earlier but lifted restrictions during Delta period in contrast to Latvia and restricted travellers from entering at the beginning of Omicron period [23], in line with the similarity of Estonia shifting between Latvia and Finland. However, as diversity was lower during Alpha and Omicron period compared with Delta, dissimilarity to Finland and Latvia may have been less well detected and higher similarity to Finland could have been merely due to larger number of people travelling between Estonia and Finland (31% of foreign visitors to Estonia, 21% of visits abroad by Estonian residents in 2021) compared with Latvia (17% and 10%, respectively) [45]. Russia implemented strict travel restrictions throughout the pandemic with a few short-term eases [23] and only 5% of travellers to or from Estonia came from or visited Russia [45], explaining high dissimilarity with St. Petersburg.

Some limitations of our study should be noted. First, we used sequences from SARS-CoV-2 infected individuals who were tested for the virus. Thus, populations seeking testing less likely were underrepresented, which may also include children who were more often asymptomatic [3] and thus less likely to go to testing. Second, only a small proportion of all samples had been sequenced which provided lineage information for this study. As a result, the number of samples was small when stratified by counties and age group. Thus, we could only include the most predominant lineages in the regression models and accurate detection of the emergence of lineages when the number of cases is small could have been hampered, influencing the results of the comparison of the spread in adults and children. Nevertheless, most of the time, the proportion of samples sequenced surpassed 0.5%—a recommended benchmark for genomic surveillance of SARS-CoV-2 and deemed to be sufficient for monitoring virus transmission and diversity [46]. Third, we did not use phylogenetic trees to get insight into the dispersal dynamics of the virus. However, due to a small number of sequences from children, the first inferred introduction would be difficult to estimate also from the phylogenetic tree [24]. Considering also that detailed mapping of dynamics of lineages also requires well-reported epidemiological data [9, 47], we took a broad approach to delineating differences between children and adults. Finally, we could not determine the reasons for the differences seen between the regions, e.g., travel restrictions, due to lack of mobility data, which is the most important factor driving the spread of infection between regions [8, 9] and omitting it from the analysis probably leads to incorrect results. As there may be disparities between lineage distributions among sequences in GISAID and those from studies where random sampling is performed, e.g., by June 2021, B.1.617.2 was the predominant lineage in St. Petersburg [48], but this was not reflected in GISAID data, we did not perform in-depth analysis of causes for varying similarity between countries.

In conclusion, the distribution of SARS-CoV-2 lineages was similar in children and adults in Estonia. The occurrence of predominant SARS-CoV-2 lineages earlier among travel-related cases and in the regions of Estonia connected the best and varying similarity with neighbouring countries over time, suggest the importance of mobility in the establishment of the spread of lineages.

## Supporting information

S1 File Supplementary figures and tables. (DOCX)

S2 File Aggregated dataset. (CSV)
