# Supplementary material for: Dynamics of SARS-CoV-2 lineages in children and adults in 2021 and 2022
Source: PLoS One. 2024 Dec 20;19(12):e0316213. doi: 10.1371/journal.pone.0316213 (PMC11661589; doi:10.1371/journal.pone.0316213)
Supplement: S1 File — (DOCX) [file pone.0316213.s001.docx]

**Table S1**. Differences in Simpson’s genetic diversity index between counties over time.

| Comparison | Time period | Difference in genetic diversity | p-value |
| --- | --- | --- | --- |
| Harju vs Tartu | 01.01.2021-30.10.2022 | -0.014 (0.013) | 0.3 |
| **Harju vs Ida-Viru** | 01.02.2021-27.06.2021  **28.06.2021-24.10.2021**  25.10.2021-30.10.2022 | -0.012 (0.032)  **0.101 (0.039)**  0.036 (0.021) | 0.7  0.012  0.092 |
| Harju vs Other | 01.01.2021-30.10.2022 | 0.003 (0.009) | 0.8 |
| **Tartu vs Ida-Viru** | 01.02.2021-23.05.2021  **24.05.2021-05.12.2021**  06.12.2021-30.10.2022 | -0.039 (0.037)  **0.196 (0.037)**  0.005 (0.021) | 0.3  **<0.001**  0.8 |
| Tartu vs Other | 01.01.2021-30.10.2022 | -0.008 (0.011) | 0.5 |
| **Ida-Viru vs Other** | **01.02.2021-23.05.2021**  **24.05.2021-19.12.2021**  20.12.2021-30.10.2022 | **0.081 (0.034)**  **-0.192 (0.034)**  -0.032 (0.020) | **0.021**  **<0.001**  0.1 |


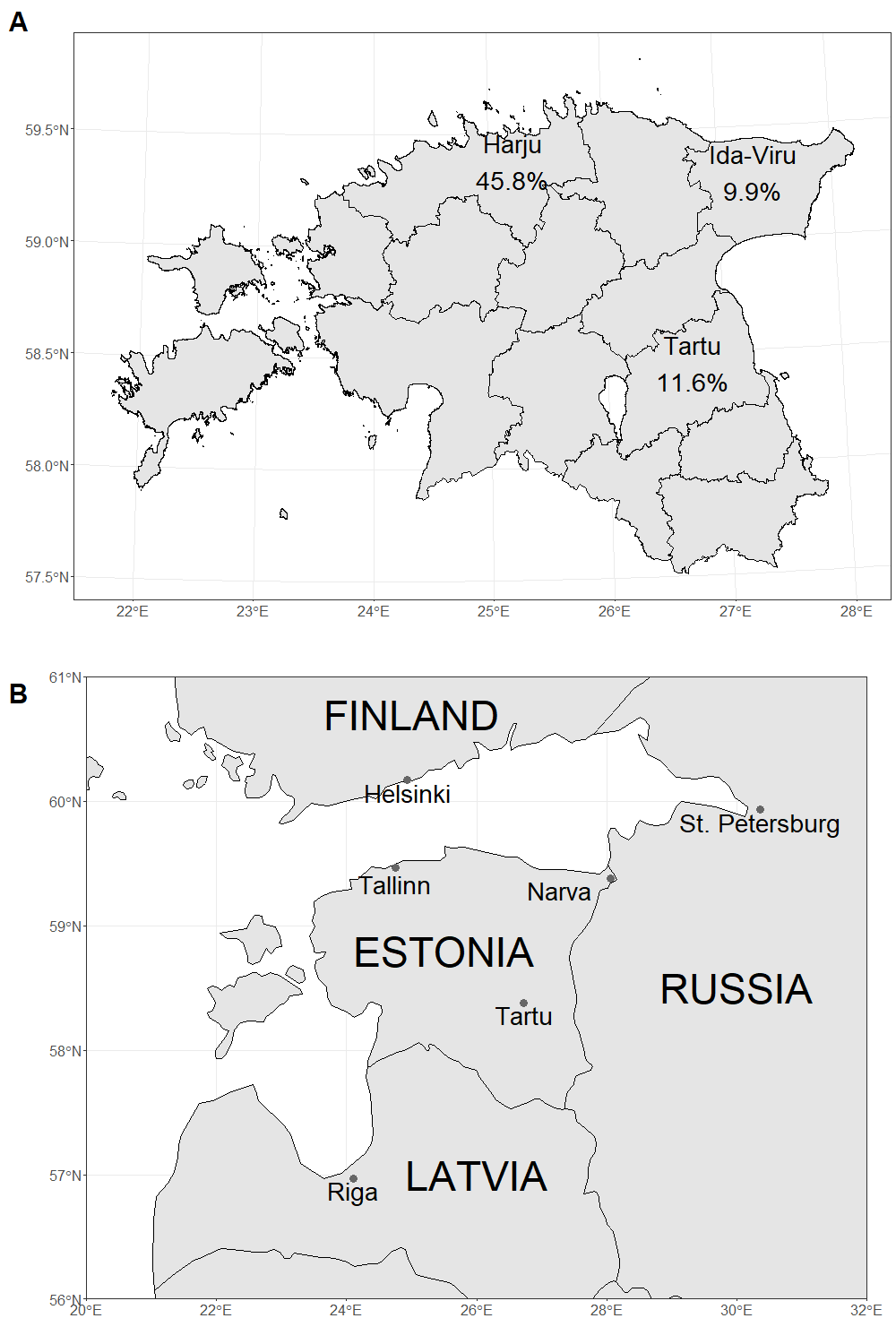


**Figure S1**. Geographical map of (A) Estonia with its county borders and three largest counties shown with the proportion of Estonian population living in these counties and (B) Estonia and its neighbouring countries with the largest cities/towns shown. Tallinn (the capital of Estonia), Tartu and Narva are the regional capitals of Harju, Tartu and Ida-Viru counties. The upper map (A) was created in R Statistical Software (version 4.3.2; R Core Team 2023) using packages sf (version 1.0-16) and ggplot2 (version 3.4.4) and shapefile (.shp) format of freely available data of administrative and settlement units on the Geoportal – Webpage of Estonian Land Board for Spatial Data (<https://geoportaal.maaamet.ee/eng/spatial-data/administrative-and-settlement-division-p312.html>; downloaded on April 19, 2024). The lower map (B) was created in R Statistical Software (version 4.3.2; R Core Team 2023) using packages ggplot2 (version 3.4.4), rnaturalearth (version 1.0.1) and rnaturalearthdata (version 1.0.0), which includes spatial data required for the figure. The towns were added to the map based on their latitude and longitude coordinates available on the LatLong.net (<https://www.latlong.net>; accessed on April 19, 2024). The upper and lower maps were arranged into one figure in R Statistical Software (version 4.3.2; R Core Team 2023) using package ggpubr (version 0.6.0).


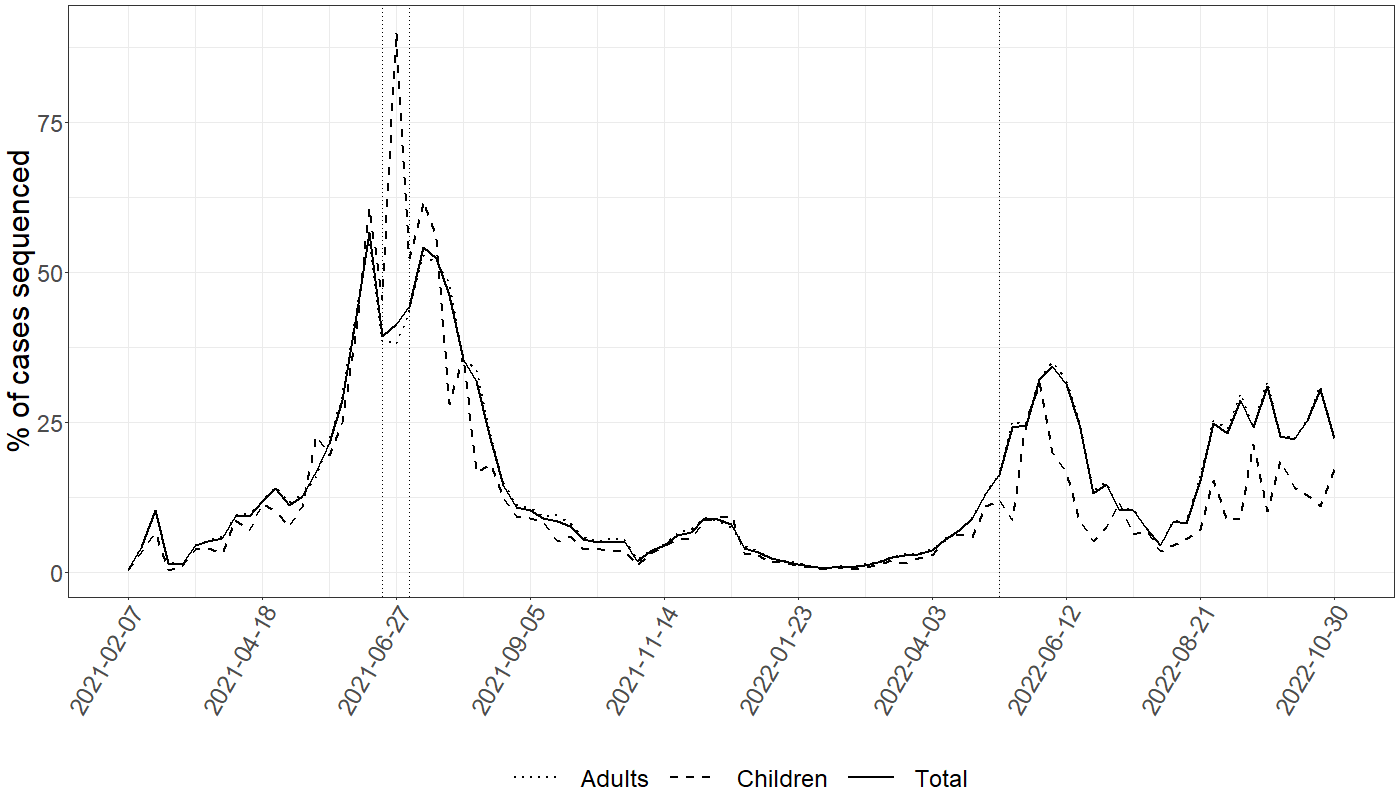


**Figure S2**. The percentage of SARS-CoV-2 PCR-positive cases in children (age <15 years), adults (age ≥15 years) and in total sequenced over time. Due to a large number of cases in adults, the total percentage largely overlaps with the proportion of cases sequenced in adults. Vertical dotted lines delineate time periods when the proportion was statistically significantly different between adults and children: from June 20 to July 4, 2021 (in children, the proportion 31.2% (95% confidence interval 22.4-40.0%) higher than in adults) and since May 8, 2022 (in children, the proportion 8% (4.4-11.6%) smaller).


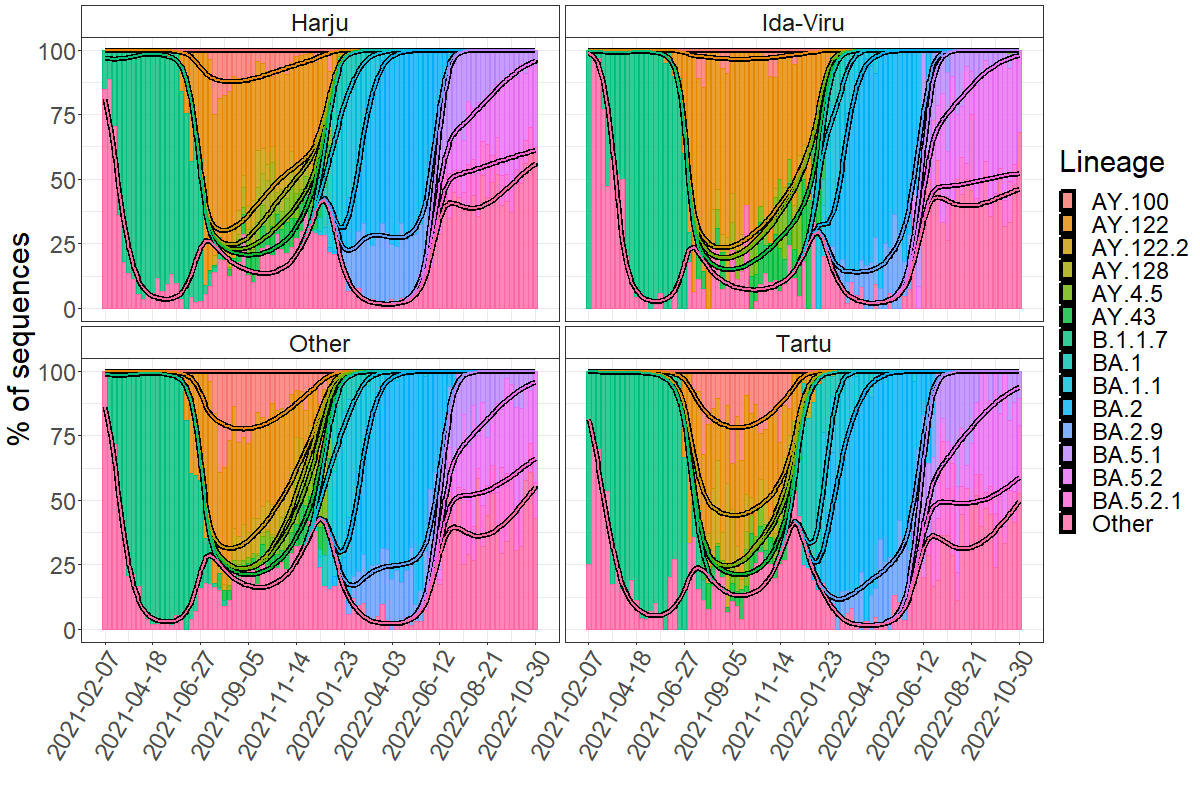


**Figure S3.** Fit of the final multinomial logistic regression of the proportions of the most common lineages over time in the three largest regions of Estonia and the other regions. Bars show the distribution of the respective lineages among sequenced samples. Lines show the predictions of the respective proportions from the model.


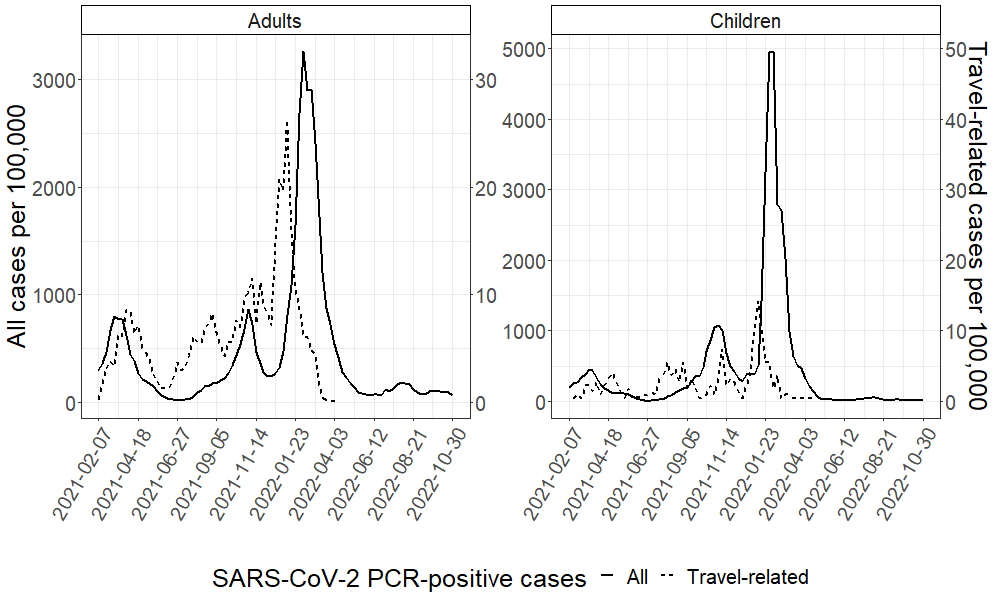


**Figure S4.** Number of sequenced SARS-CoV-2 PCR-positive cases per 100,000 reported by Estonian Health Board (solid line) and travel-related cases per 100,000 (dashed lines) in adults and children. All travel-related cases were sequenced except between February 1-6, 2021, and January 6-15, 2022, when 27% and 41% of all cases, respectively, were sequenced.


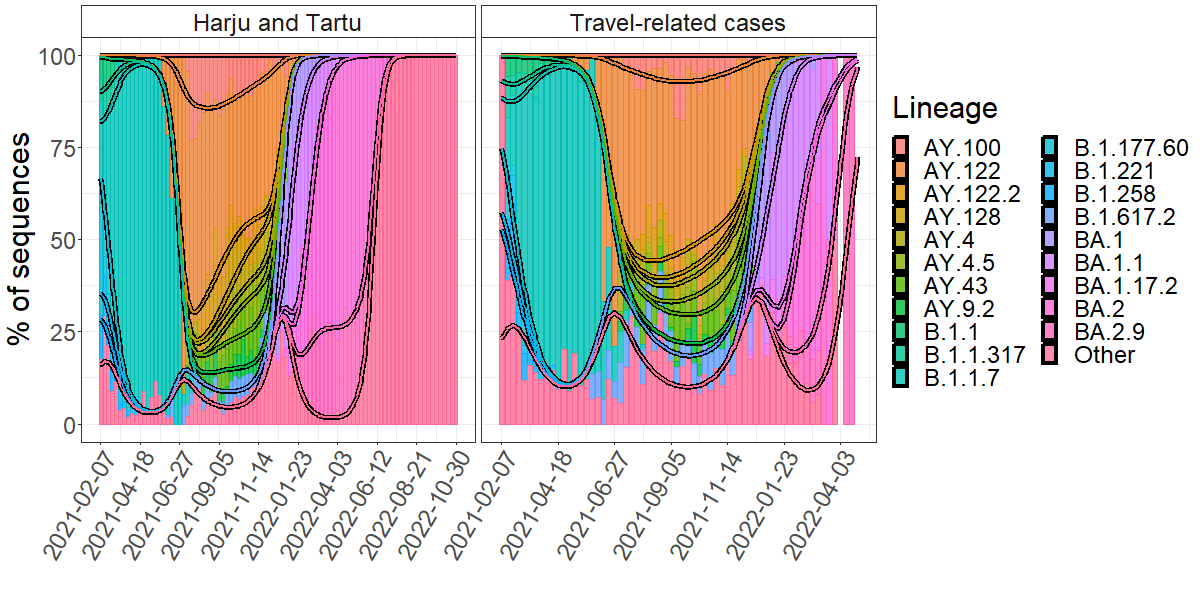


**Figure S5**. Fit of the final multinomial logistic regression of the proportions of the most common lineages over time among travel-related cases and the two largest regions of Estonia. Bars show the distribution of the respective lineages among sequenced samples. Lines show the predictions of the respective proportions from the model.


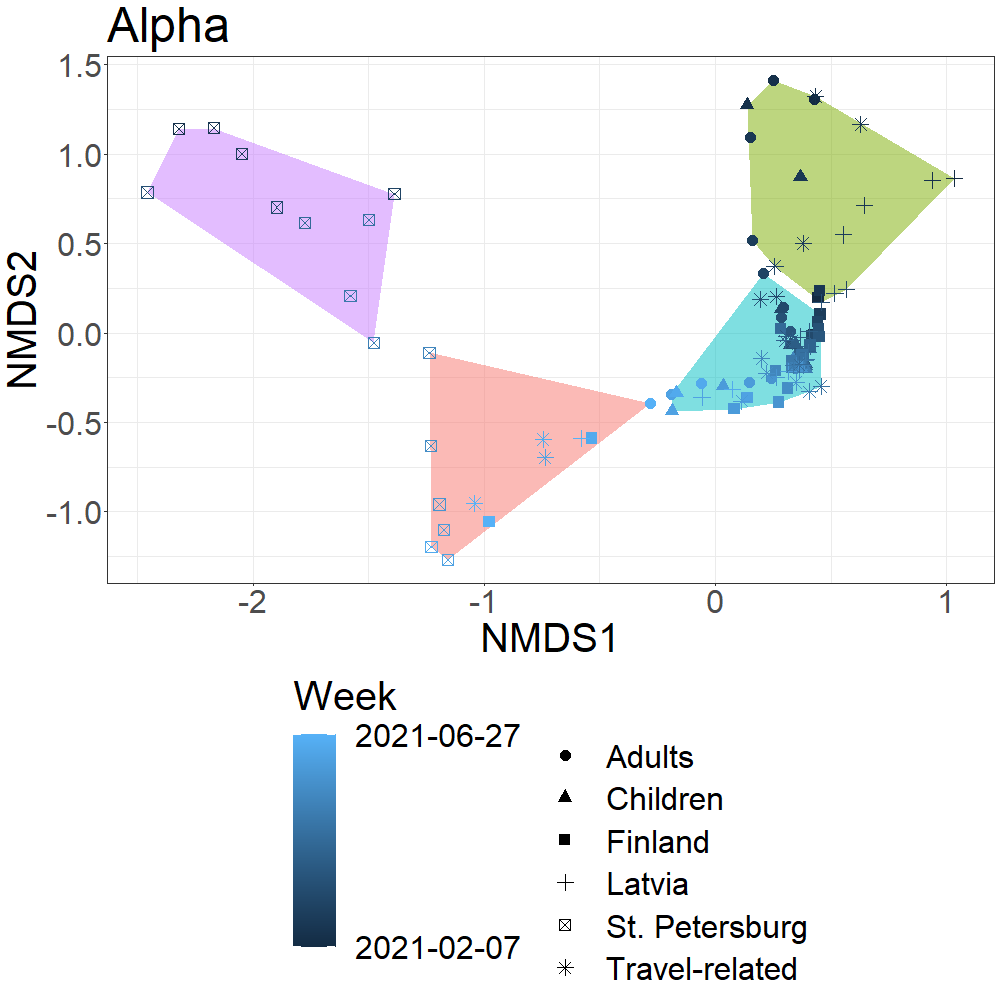


**Figure S6**. The plot of the two first dimensions of non-metric multidimensional scaling of Bray-Curtis dissimilarity matrix between Estonian adults, children and travel-related cases and Finland, Latvia and St. Petersburg between 01.02.2021-27.06.2021 when Alpha lineages comprised at least 50% of all samples in Estonia. The colouring of the data points shows the time - darker points show earlier and lighter points later weeks. Polygons show clusters of the points which membership degrees were highest for a particular cluster.


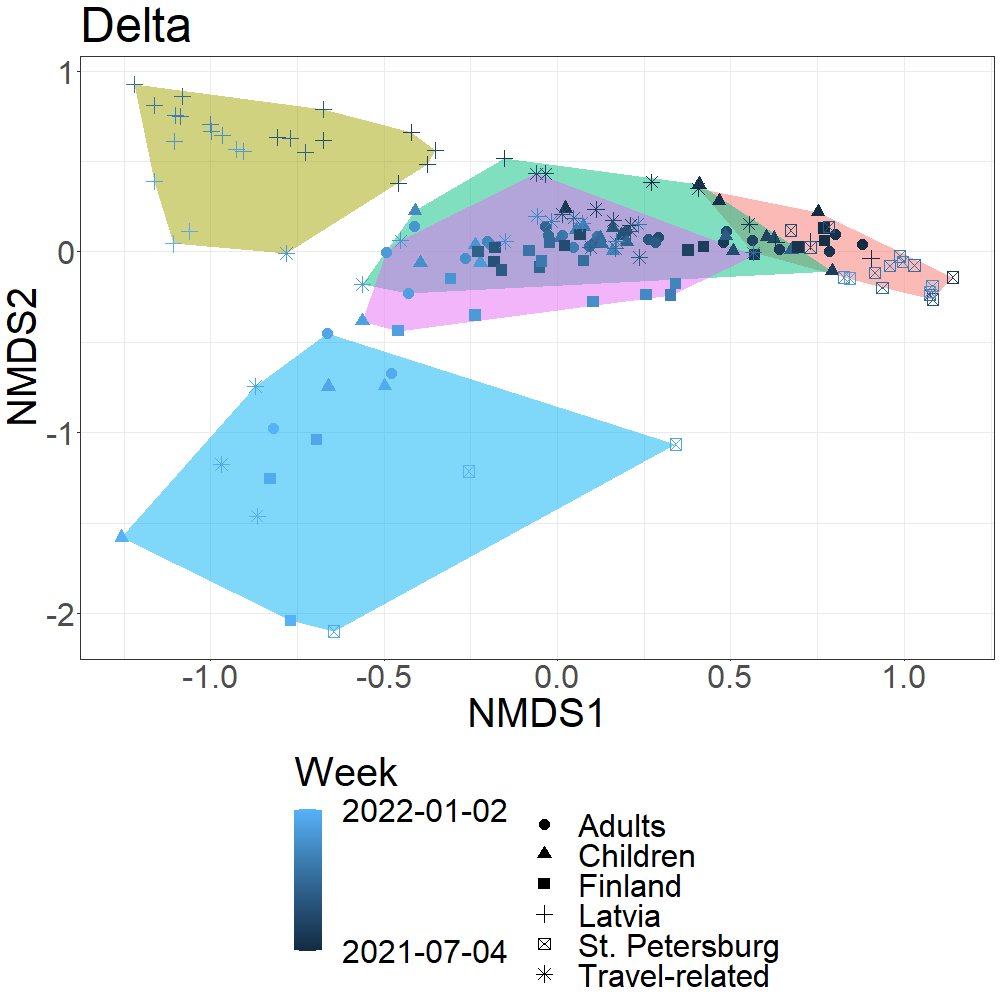


**Figure S7**. The plot of the two first dimensions of non-metric multidimensional scaling of Bray-Curtis dissimilarity matrix between Estonian adults, children and travel-related cases and Finland, Latvia and St. Petersburg between 28.06.2021-02.01.2022 when Delta lineages comprised at least 50% of all samples in Estonia. The colouring of the data points shows the time - darker points show earlier and lighter points later weeks. Polygons show clusters of the points which membership degrees were highest for a particular cluster.


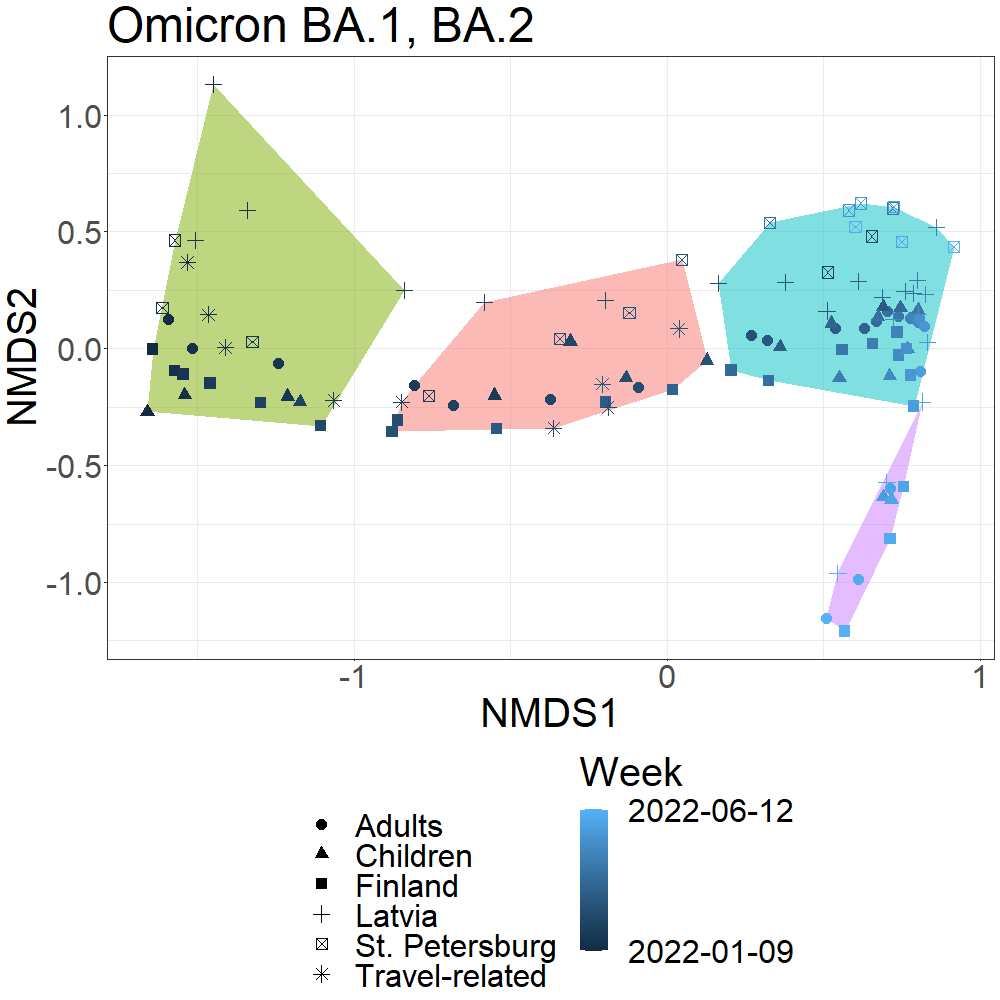


**Figure S8**. The plot of the two first dimensions of non-metric multidimensional scaling of Bray-Curtis dissimilarity matrix between Estonian adults, children and travel-related cases and Finland, Latvia and St. Petersburg between 03.01.2022-12.06.2022 when Omicron BA.1 and BA.2 lineages comprised at least 50% of all samples in Estonia. The colouring of the data points shows the time - darker points show earlier and lighter points later weeks. Polygons show clusters of the points which membership degrees were highest for a particular cluster.


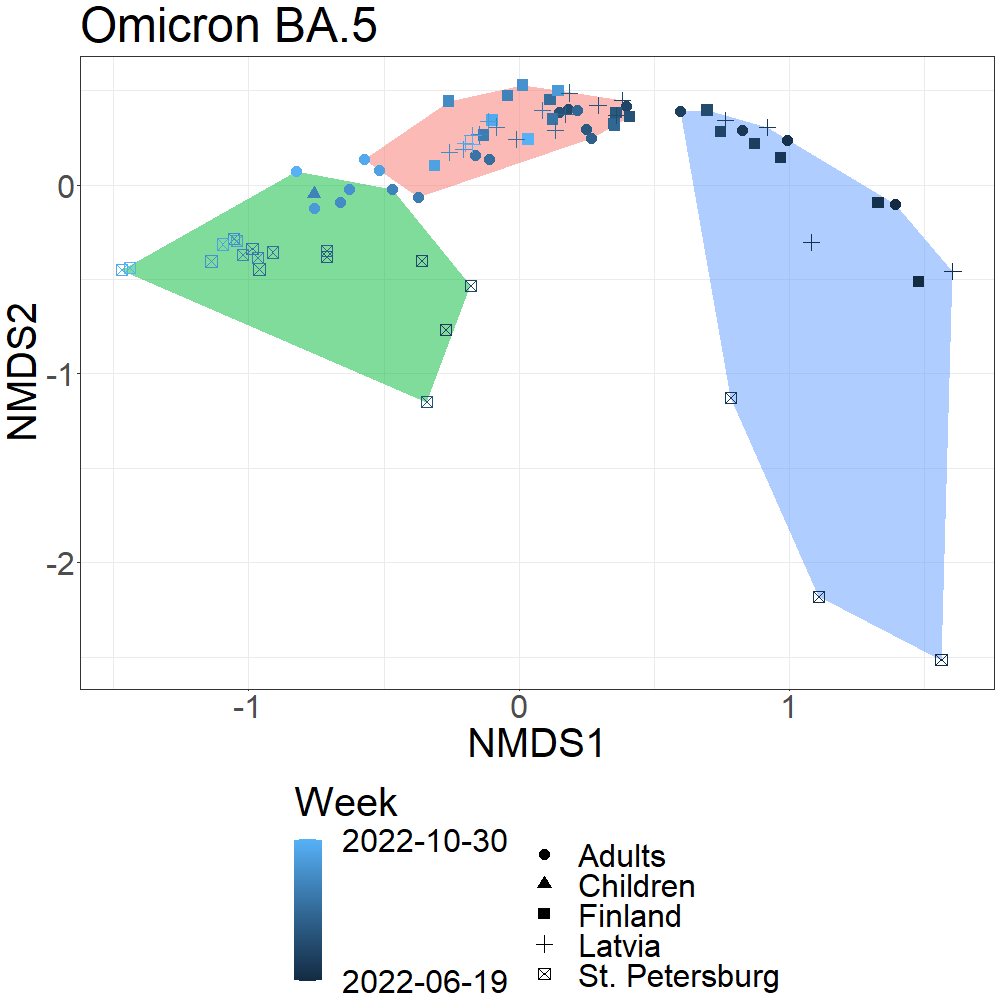


**Figure S9**. The plot of the two first dimensions of non-metric multidimensional scaling of Bray-Curtis dissimilarity matrix between Estonian adults and children and Finland, Latvia and St. Petersburg between 13.06.2022-30.10.2022 when Omicron BA.5 lineages comprised at least 50% of all samples in Estonia. The colouring of the data points shows the time - darker points show earlier and lighter points later weeks. Polygons show clusters of the points which membership degrees were highest for a particular cluster.


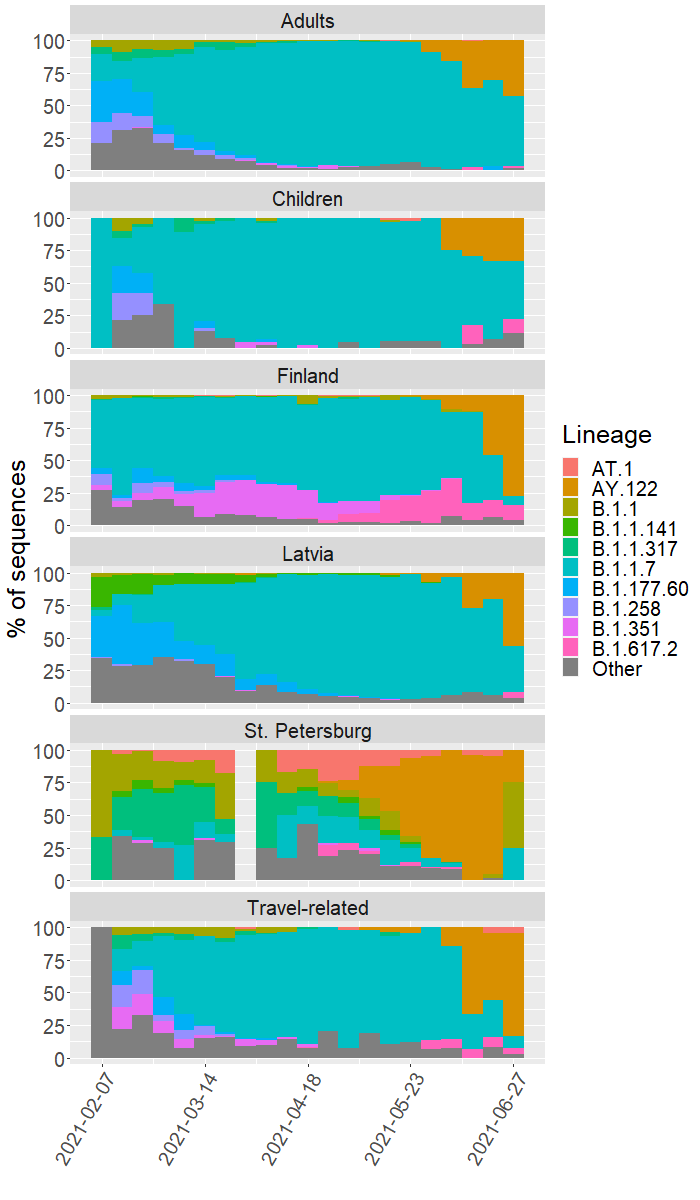


**Figure S10**. Weekly percentages of the most common lineages Estonian adults, children and travel-related cases, Finland, Latvia and St. Petersburg during Alpha variant predominance period. The lineages that comprised >20% in at least one week with at least ten samples in at least one country are shown.


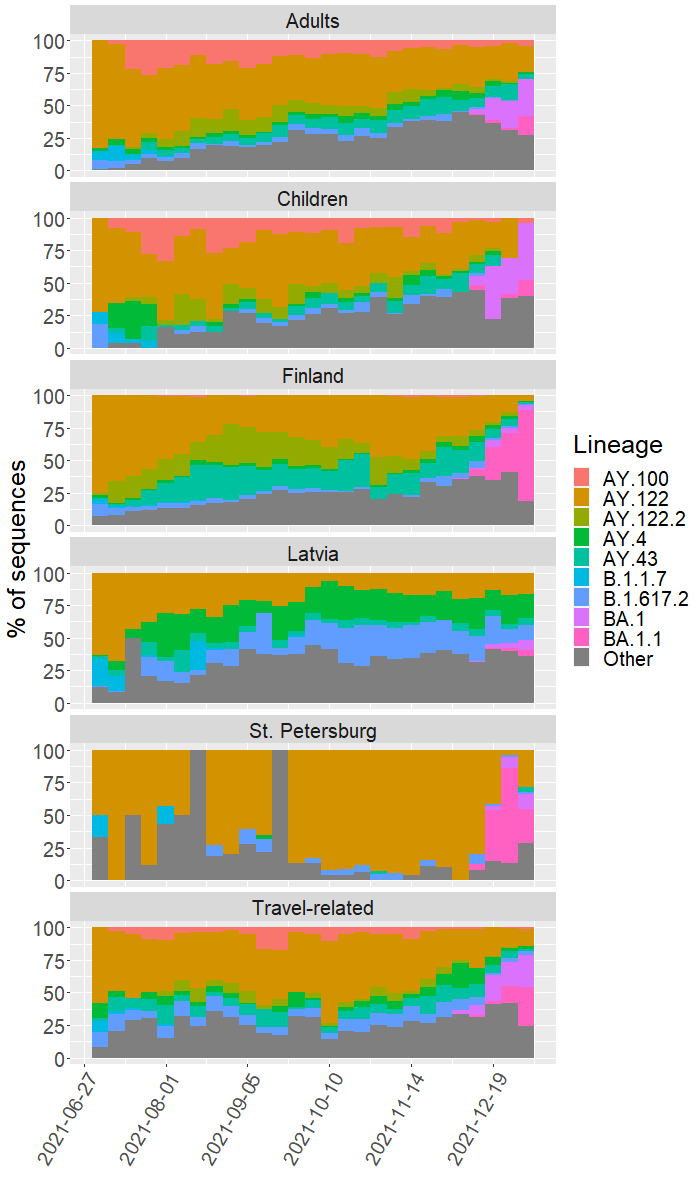


**Figure S11**. Weekly percentages of the most common lineages in Estonian adults, children and travel-related cases, Finland, Latvia and St. Petersburg during Delta variant predominance period. The lineages that comprised >20% in at least one week with at least ten samples in at least one country are shown.


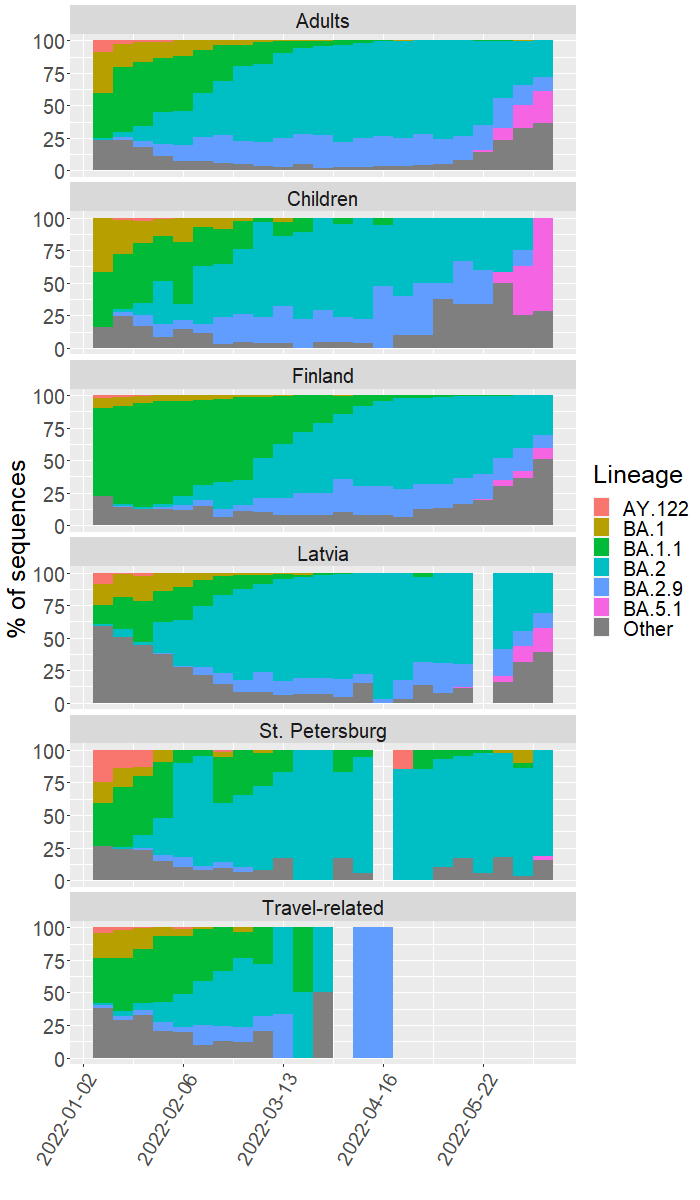


**Figure S12**. Weekly percentages of the most common lineages in Estonian adults, children, Finland, Latvia and St. Petersburg during Omicron BA.1 and BA.2 variant predominance period. The lineages that comprised >20% in at least one week with at least ten samples in at least one country are shown.


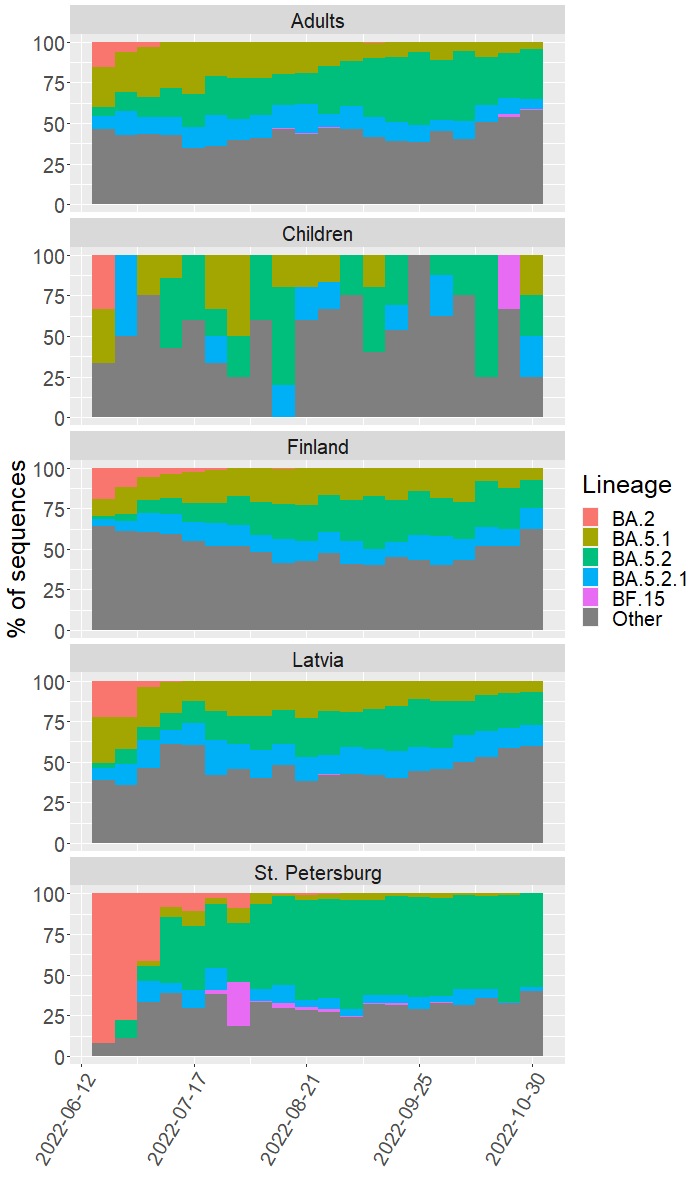


**Figure S13**. Weekly percentages of the most common lineages in Estonian adults, children and travel-related cases, Finland, Latvia and St. Petersburg during Omicron BA.5 variant predominance period. The lineages that comprised >20% in at least one week with at least ten samples in at least one country are shown.
